# Supplementary material for: Structural Investigation of Orf Virus Bcl-2 Homolog ORFV125 Interactions with BH3-Motifs from BH3-Only Proteins Puma and Hrk
Source: Viruses. 2021 Jul 15;13(7):1374. doi: 10.3390/v13071374 (PMC8310162; doi:10.3390/v13071374)
Supplement: Supplementary file 1 [file viruses-13-01374-s001.zip › viruses-1268202-si.pdf]

Preliminary Full wwPDB X-ray Structure Validation Report (i)

Jun 30, 2021 – 03:26 pm BST

Deposition ID : D_1292116246

This is a Preliminary Full wwPDB X-ray Structure Validation Report.

This report is produced by the wwPDB Deposition System during initial deposition but before annotation of the structure.

We welcome your comments at *validation@mail.wwpdb.org*
A user guide is available at
https://www.wwpdb.org/validation/2017/XrayValidationReportHelp
with specific help available everywhere you see the (i) symbol.

The following versions of software and data (see references (i)) were used in the production of this report:

MolProbity	:	4.02b-467
Mogul	:	1.8.5 (274361), CSD as541be (2020)
Xtriage (Phenix)	:	1.13
EDS	:	2.22
Percentile statistics	:	20191225.v01 (using entries in the PDB archive December 25th 2019)
Refmac	:	5.8.0158
CCP4	:	7.0.044 (Gargrove)
Ideal geometry (proteins)	:	Engh & Huber (2001)
Ideal geometry (DNA, RNA)	:	Parkinson et al. (1996)
Validation Pipeline (wwPDB-VP)	:	2.22

1 Overall quality at a glance (i)

The following experimental techniques were used to determine the structure:
X-RAY DIFFRACTION

The reported resolution of this entry is 1.99 Å.

Percentile scores (ranging between 0-100) for global validation metrics of the entry are shown in the following graphic. The table shows the number of entries on which the scores are based.

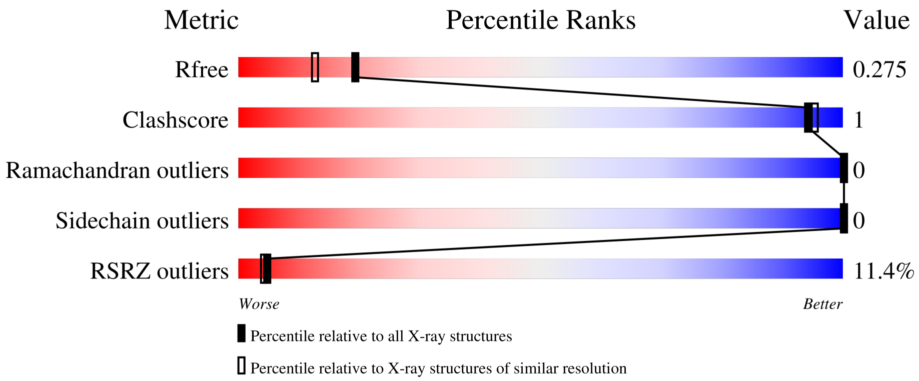

Metric	Whole archive (#Entries)	Similar resolution (#Entries, resolution range(Å))
R_{free}	130704	8085 (2.00-2.00)
Clashscore	141614	9178 (2.00-2.00)
Ramachandran outliers	138981	9054 (2.00-2.00)
Sidechain outliers	138945	9053 (2.00-2.00)
RSRZ outliers	127900	7900 (2.00-2.00)

The table below summarises the geometric issues observed across the polymeric chains and their fit to the electron density. The red, orange, yellow and green segments of the lower bar indicate the fraction of residues that contain outliers for >=3, 2, 1 and 0 types of geometric quality criteria respectively. A grey segment represents the fraction of residues that are not modelled. The numeric value for each fraction is indicated below the corresponding segment, with a dot representing fractions <=5% The upper red bar (where present) indicates the fraction of residues that have poor fit to the electron density. The numeric value is given above the bar.

Mol	Chain	Length	Quality of chain
1	A	136	6% / 97% •
2	U	26	4% / 96% •
3	B	132	7% / 94% 6%
4	C	24	8% / 96% •
5	D	128	14% / 98% •

Continued on next page...

Continued from previous page...

Mol	Chain	Length	Quality of chain
6	E	22	18% / 100%
7	F	129	17% / 97% •
8	G	21	29% / 100%

2 Entry composition (i)

There are 11 unique types of molecules in this entry. The entry contains 9641 atoms, of which 4772 are hydrogens and 0 are deuteriums.

In the tables below, the ZeroOcc column contains the number of atoms modelled with zero occupancy, the AltConf column contains the number of residues with at least one atom in alternate conformation and the Trace column contains the number of residues modelled with at most 2 atoms.

- Molecule 1 is a protein.

Mol	Chain	Residues	Atoms						ZeroOcc	AltConf	Trace
			Total	C	H	N	O	S			
1	A	136	2073	651	1025	186	206	5	0	0	0

- Molecule 2 is a protein.

Mol	Chain	Residues	Atoms						ZeroOcc	AltConf	Trace
			Total	C	H	N	O	S			
2	U	26	419	126	212	44	36	1	0	0	0

- Molecule 3 is a protein.

Mol	Chain	Residues	Atoms						ZeroOcc	AltConf	Trace
			Total	C	H	N	O	S			
3	B	132	2033	638	1011	183	196	5	0	0	0

- Molecule 4 is a protein called SER-ALA-ALA-GLN-LEU-THR-ALA-ALA-ARG-LEU-LYS-ALA-LEU-GLY-ASP-GLU-LEU-HIS-GLN-ARG-THR-MET-TRP-ARG.

Mol	Chain	Residues	Atoms						ZeroOcc	AltConf	Trace
			Total	C	H	N	O	S			
4	C	24	384	117	194	39	33	1	0	0	0

- Molecule 5 is a protein.

Mol	Chain	Residues	Atoms						ZeroOcc	AltConf	Trace
			Total	C	H	N	O	S			
5	D	128	1967	619	978	175	190	5	0	0	0

- Molecule 6 is a protein called SER-ALA-ALA-GLN-LEU-THR-ALA-ALA-ARG-LEU-LYS-ALA-LEU-GLY-ASP-GLU-LEU-HIS-GLN-ARG-THR-MET.

Mol	Chain	Residues	Atoms						ZeroOcc	AltConf	Trace
			Total	C	H	N	O	S			
6	E	22	336	100	171	33	31	1	0	0	0

- Molecule 7 is a protein.

Mol	Chain	Residues	Atoms						ZeroOcc	AltConf	Trace
			Total	C	H	N	O	S			
7	F	129	1972	619	979	178	191	5	0	0	0

- Molecule 8 is a protein called SER-ALA-ALA-GLN-LEU-THR-ALA-ALA-ARG-LEU-LYS-ALA-LEU-GLY-ASP-GLU-LEU-HIS-GLN-ARG-THR.

Mol	Chain	Residues	Atoms					ZeroOcc	AltConf	Trace
			Total	C	H	N	O			
8	G	21	319	95	162	32	30	0	0	0

- Molecule 9 is 1,2-ETHANEDIOL (three-letter code: EDO) (formula: $C_2H_6O_2$).

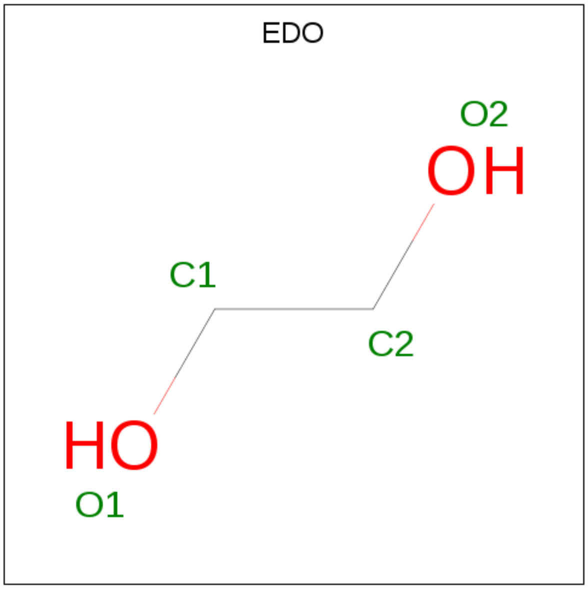

Mol	Chain	Residues	Atoms				ZeroOcc	AltConf
			Total	C	H	O		
9	A	1	10	2	6	2	0	0
9	A	1	10	2	6	2	0	0
9	F	1	10	2	6	2	0	0
9	F	1	10	2	6	2	0	0

- Molecule 10 is GLYCEROL (three-letter code: GOL) (formula: $C_3H_8O_3$).

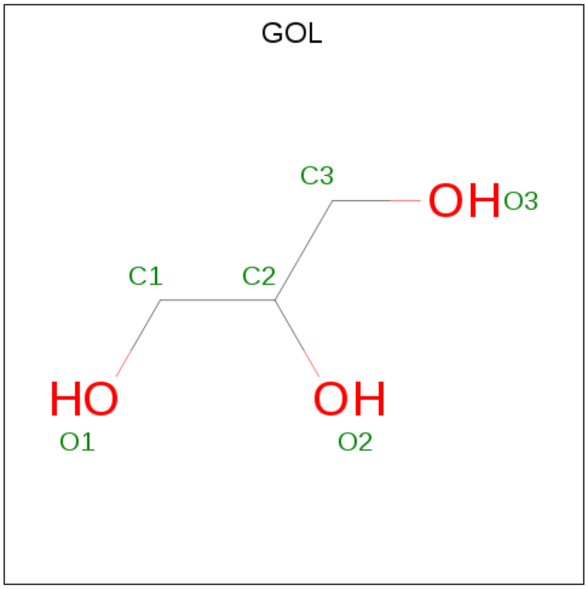

Mol	Chain	Residues	Atoms	ZeroOcc	AltConf
10	A	1	Total C H O<br>14 3 8 3	0	0
10	C	1	Total C H O<br>14 3 8 3	0	0

- Molecule 11 is water.

Mol	Chain	Residues	Atoms	ZeroOcc	AltConf
11	S	70	Total O<br>70 70	0	0

3 Residue-property plots (i)

These plots are drawn for all protein, RNA, DNA and oligosaccharide chains in the entry. The first graphic for a chain summarises the proportions of the various outlier classes displayed in the second graphic. The second graphic shows the sequence view annotated by issues in geometry and electron density. Residues are color-coded according to the number of geometric quality criteria for which they contain at least one outlier: green = 0, yellow = 1, orange = 2 and red = 3 or more. A red dot above a residue indicates a poor fit to the electron density (RSRZ > 2). Stretches of 2 or more consecutive residues without any outlier are shown as a green connector. Residues present in the sample, but not in the model, are shown in grey.

- Molecule 1:

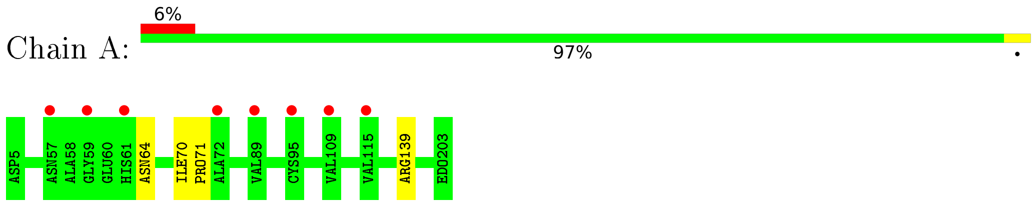

- Molecule 2:

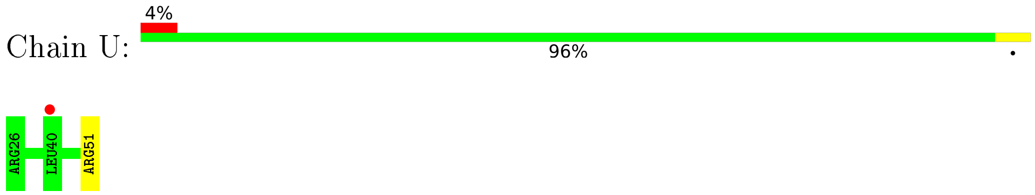

- Molecule 3:

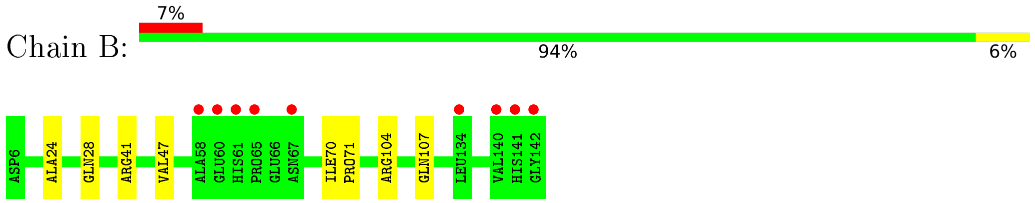

- Molecule 4: SER-ALA-ALA-GLN-LEU-THR-ALA-ALA-ARG-LEU-LYS-ALA-LEU-GLY-ASP-GLU-LEU-HIS-GLN-ARG-THR-MET-TRP-ARG

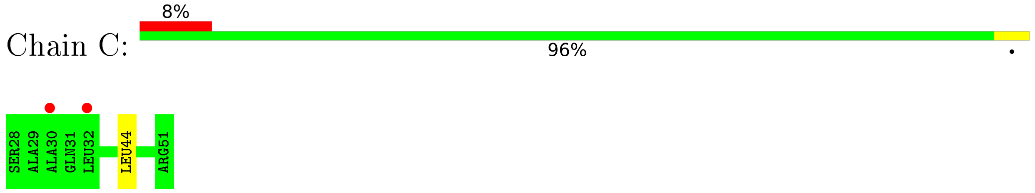

- Molecule 5:

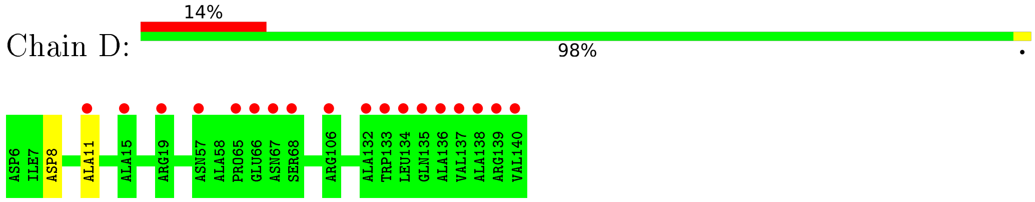

• Molecule 6: SER-ALA-ALA-GLN-LEU-THR-ALA-ALA-ARG-LEU-LYS-ALA-LEU-GLY-ASP-GLU-LEU-HIS-GLN-ARG-THR-MET

Chain E:

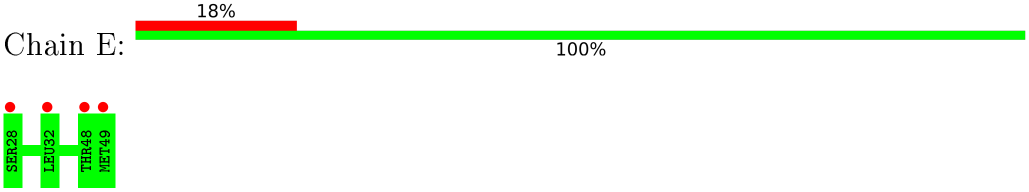

• Molecule 7:

Chain F:

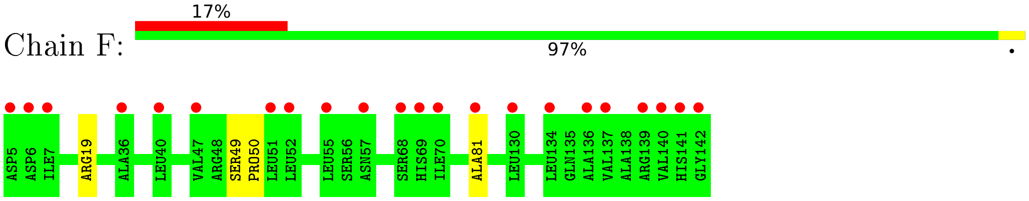

• Molecule 8: SER-ALA-ALA-GLN-LEU-THR-ALA-ALA-ARG-LEU-LYS-ALA-LEU-GLY-ASP-GLU-LEU-HIS-GLN-ARG-THR

Chain G:

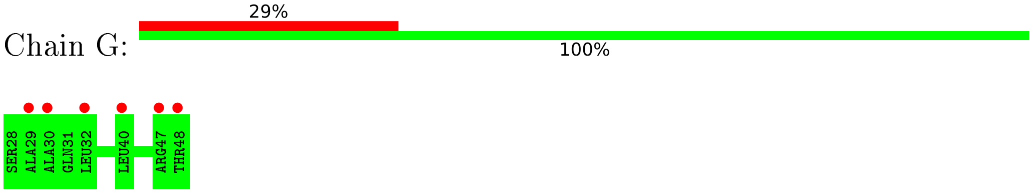

4 Data and refinement statistics (i)

Property	Value	Source
Space group	P 1	Depositor
Cell constants<br>a, b, c, α, β, γ	46.41Å 57.70Å 65.06Å<br>71.88° 76.61° 75.08°	Depositor
Resolution (Å)	38.57 – 1.99<br>38.57 – 1.99	Depositor<br>EDS
% Data completeness<br>(in resolution range)	95.9 (38.57-1.99)<br>95.9 (38.57-1.99)	Depositor<br>EDS
R_{merge}	(Not available)	Depositor
R_{sym}	(Not available)	Depositor
$< I/\sigma(I) >$ [1]	1.03 (at 2.00Å)	Xtriage
Refinement program	phenix.refine 1.11.1_2575, PHENIX 1.11.1_2575	Depositor
R, R_{free}	0.225 , 0.274<br>0.225 , 0.275	Depositor<br>DCC
R_{free} test set	1959 reflections (4.90%)	wwPDB-VP
Wilson B-factor (Å^2)	47.7	Xtriage
Anisotropy	0.219	Xtriage
Bulk solvent k_{sol}(e/Å^3), B_{sol}(Å^2)	0.39 , 58.8	EDS
L-test for twinning[2]	$< \|L\| > = 0.52$, $< L^2 > = 0.35$	Xtriage
Estimated twinning fraction	No twinning to report.	Xtriage
F_o,F_c correlation	0.96	EDS
Total number of atoms	9641	wwPDB-VP
Average B, all atoms (Å^2)	75.0	wwPDB-VP

Xtriage's analysis on translational NCS is as follows: *The largest off-origin peak in the Patterson function is 8.31% of the height of the origin peak. No significant pseudotranslation is detected.*

[1] Intensities estimated from amplitudes.

[2] Theoretical values of $< |L| >$, $< L^2 >$ for acentric reflections are 0.5, 0.333 respectively for untwinned datasets, and 0.375, 0.2 for perfectly twinned datasets.

5 Model quality (i)

5.1 Standard geometry (i)

Bond lengths and bond angles in the following residue types are not validated in this section: GOL, EDO

The Z score for a bond length (or angle) is the number of standard deviations the observed value is removed from the expected value. A bond length (or angle) with $|Z| > 5$ is considered an outlier worth inspection. RMSZ is the root-mean-square of all Z scores of the bond lengths (or angles).

Mol	Chain	Bond lengths		Bond angles	
		RMSZ	#|Z| >5	RMSZ	#|Z| >5
1	A	0.36	0/1054	0.50	0/1432
2	U	0.36	0/209	0.49	0/279
3	B	0.35	0/1037	0.51	0/1406
4	C	0.28	0/192	0.45	0/257
5	D	0.34	0/1003	0.49	0/1362
6	E	0.34	0/165	0.50	0/220
7	F	0.30	0/1007	0.46	0/1369
8	G	0.25	0/157	0.37	0/210
All	All	0.33	0/4824	0.49	0/6535

Chiral center outliers are detected by calculating the chiral volume of a chiral center and verifying if the center is modelled as a planar moiety or with the opposite hand.A planarity outlier is detected by checking planarity of atoms in a peptide group, atoms in a mainchain group or atoms of a sidechain that are expected to be planar.

Mol	Chain	#Chirality outliers	#Planarity outliers
7	F	0	1

There are no bond length outliers.

There are no bond angle outliers.

There are no chirality outliers.

All (1) planarity outliers are listed below:

Mol	Chain	Res	Type	Group
7	F	81	ALA	Peptide

5.2 Too-close contacts (i)

In the following table, the Non-H and H(model) columns list the number of non-hydrogen atoms and hydrogen atoms in the chain respectively. The H(added) column lists the number of hydrogen atoms added and optimized by MolProbity. The Clashes column lists the number of clashes within the asymmetric unit, whereas Symm-Clashes lists symmetry-related clashes.

Mol	Chain	Non-H	H(model)	H(added)	Clashes	Symm-Clashes
1	A	1048	1025	1038	3	0
2	U	207	212	212	1	0
3	B	1022	1011	1014	7	0
4	C	190	194	194	2	0
5	D	989	978	992	1	0
6	E	165	171	171	0	0
7	F	993	979	990	2	0
8	G	157	162	162	0	0
9	A	8	12	12	0	0
9	F	8	12	12	0	0
10	A	6	8	8	0	0
10	C	6	8	8	0	0
11	S	70	0	0	1	0
All	All	4869	4772	4813	12	0

The all-atom clashscore is defined as the number of clashes found per 1000 atoms (including hydrogen atoms). The all-atom clashscore for this structure is 1.

All (12) close contacts within the same asymmetric unit are listed below, sorted by their clash magnitude.

Atom-1	Atom-2	Interatomic distance (Å)	Clash overlap (Å)
1:A:139:ARG:NH1	2:U:51:ARG:O	2.24	0.70
1:A:64:ASN:N	11:S:79:HOH:O	2.38	0.56
5:D:8:ASP:OD2	5:D:11:ALA:N	2.37	0.53
3:B:47:VAL:HG21	4:C:44:LEU:HD22	1.90	0.52
3:B:70:ILE:HB	3:B:71:PRO:HD3	1.91	0.51
3:B:47:VAL:O	3:B:47:VAL:HG12	2.16	0.46
3:B:24:ALA:O	3:B:28:GLN:HG2	2.18	0.44
7:F:49:SER:HB2	7:F:50:PRO:HD3	2.00	0.43
3:B:47:VAL:CG2	4:C:44:LEU:HD22	2.48	0.42
1:A:70:ILE:HB	1:A:71:PRO:HD3	2.00	0.42
3:B:41:ARG:NH1	7:F:19:ARG:HD3	2.36	0.41
3:B:104:ARG:O	3:B:107:GLN:HG2	2.22	0.40

There are no symmetry-related clashes.

5.3 Torsion angles ⓘ

5.3.1 Protein backbone ⓘ

In the following table, the Percentiles column shows the percent Ramachandran outliers of the chain as a percentile score with respect to all X-ray entries followed by that with respect to entries of similar resolution.

The Analysed column shows the number of residues for which the backbone conformation was analysed, and the total number of residues.

Mol	Chain	Analysed	Favoured	Allowed	Outliers	Percentiles	
1	A	128/136 (94%)	124 (97%)	4 (3%)	0	100	100
2	U	24/26 (92%)	24 (100%)	0	0	100	100
3	B	124/132 (94%)	119 (96%)	5 (4%)	0	100	100
4	C	22/24 (92%)	22 (100%)	0	0	100	100
5	D	122/128 (95%)	120 (98%)	2 (2%)	0	100	100
6	E	20/22 (91%)	20 (100%)	0	0	100	100
7	F	125/129 (97%)	121 (97%)	4 (3%)	0	100	100
8	G	19/21 (90%)	19 (100%)	0	0	100	100
All	All	584/618 (94%)	569 (97%)	15 (3%)	0	100	100

There are no Ramachandran outliers to report.

5.3.2 Protein sidechains ⓘ

In the following table, the Percentiles column shows the percent sidechain outliers of the chain as a percentile score with respect to all X-ray entries followed by that with respect to entries of similar resolution.

The Analysed column shows the number of residues for which the sidechain conformation was analysed, and the total number of residues.

Mol	Chain	Analysed	Rotameric	Outliers	Percentiles	
1	A	109/109 (100%)	109 (100%)	0	100	100
2	U	20/20 (100%)	20 (100%)	0	100	100
3	B	107/107 (100%)	107 (100%)	0	100	100
4	C	18/18 (100%)	18 (100%)	0	100	100
5	D	104/104 (100%)	104 (100%)	0	100	100
6	E	16/16 (100%)	16 (100%)	0	100	100

Continued on next page...

Continued from previous page...

Mol	Chain	Analysed	Rotameric	Outliers	Percentiles	
7	F	103/103 (100%)	103 (100%)	0	100	100
8	G	15/15 (100%)	15 (100%)	0	100	100
All	All	492/492 (100%)	492 (100%)	0	100	100

There are no protein residues with a non-rotameric sidechain to report.

Sometimes sidechains can be flipped to improve hydrogen bonding and reduce clashes. There are no such sidechains identified.

5.3.3 RNA (i)

There are no RNA molecules in this entry.

5.4 Non-standard residues in protein, DNA, RNA chains (i)

There are no non-standard protein/DNA/RNA residues in this entry.

5.5 Carbohydrates (i)

There are no monosaccharides in this entry.

5.6 Ligand geometry (i)

6 ligands are modelled in this entry.

In the following table, the Counts columns list the number of bonds (or angles) for which Mogul statistics could be retrieved, the number of bonds (or angles) that are observed in the model and the number of bonds (or angles) that are defined in the Chemical Component Dictionary. The Link column lists molecule types, if any, to which the group is linked. The Z score for a bond length (or angle) is the number of standard deviations the observed value is removed from the expected value. A bond length (or angle) with $|Z| > 2$ is considered an outlier worth inspection. RMSZ is the root-mean-square of all Z scores of the bond lengths (or angles).

Mol	Type	Chain	Res	Link	Bond lengths			Bond angles		
					Counts	RMSZ	$\#\|Z\| > 2$	Counts	RMSZ	$\#\|Z\| > 2$
9	EDO	A	205	-	3,3,3	0.48	0	2,2,2	0.26	0
10	GOL	C	101	-	5,5,5	0.39	0	5,5,5	0.19	0
10	GOL	A	206	-	5,5,5	0.29	0	5,5,5	0.42	0
9	EDO	F	202	-	3,3,3	0.47	0	2,2,2	0.22	0
9	EDO	F	201	-	3,3,3	0.44	0	2,2,2	0.29	0
9	EDO	A	204	-	3,3,3	0.55	0	2,2,2	0.15	0

In the following table, the Chirals column lists the number of chiral outliers, the number of chiral centers analysed, the number of these observed in the model and the number defined in the Chemical Component Dictionary. Similar counts are reported in the Torsion and Rings columns. '-' means no outliers of that kind were identified.

Mol	Type	Chain	Res	Link	Chirals	Torsions	Rings
9	EDO	A	205	-	-	0/1/1/1	-
10	GOL	C	101	-	-	2/4/4/4	-
10	GOL	A	206	-	-	2/4/4/4	-
9	EDO	F	202	-	-	0/1/1/1	-
9	EDO	F	201	-	-	0/1/1/1	-
9	EDO	A	204	-	-	0/1/1/1	-

There are no bond length outliers.

There are no bond angle outliers.

There are no chirality outliers.

All (4) torsion outliers are listed below:

Mol	Chain	Res	Type	Atoms
10	A	206	GOL	O1-C1-C2-C3
10	C	101	GOL	C1-C2-C3-O3
10	A	206	GOL	O1-C1-C2-O2
10	C	101	GOL	O2-C2-C3-O3

There are no ring outliers.

No monomer is involved in short contacts.

5.7 Other polymers (i)

There are no such residues in this entry.

5.8 Polymer linkage issues (i)

The following chains have linkage breaks:

Mol	Chain	Number of breaks
3	B	3
5	D	2
1	A	2
7	F	1

All chain breaks are listed below:

Model	Chain	Residue-1	Atom-1	Residue-2	Atom-2	Distance (Å)
1	F	58:ALA	C	67:ASN	N	13.85
1	D	58:ALA	C	65:PRO	N	7.42
1	A	61:HIS	C	64:ASN	N	6.55
1	B	58:ALA	C	60:GLU	N	5.59
1	B	61:HIS	C	65:PRO	N	5.23
1	D	80:GLU	C	82:PRO	N	3.53
1	A	80:GLU	C	82:PRO	N	3.48
1	B	80:GLU	C	82:PRO	N	3.46

6 Fit of model and data (i)

6.1 Protein, DNA and RNA chains (i)

In the following table, the column labelled '#RSRZ> 2' contains the number (and percentage) of RSRZ outliers, followed by percent RSRZ outliers for the chain as percentile scores relative to all X-ray entries and entries of similar resolution. The OWAB column contains the minimum, median, 95th percentile and maximum values of the occupancy-weighted average B-factor per residue. The column labelled 'Q< 0.9' lists the number of (and percentage) of residues with an average occupancy less than 0.9.

Mol	Chain	Analysed	<RSRZ>	#RSRZ>2			OWAB(Å^2)	Q<0.9
1	A	134/136 (98%)	0.65	8 (5%)	21	20	37, 53, 112, 140	0
2	U	26/26 (100%)	0.68	1 (3%)	40	39	42, 51, 94, 102	0
3	B	132/132 (100%)	0.59	9 (6%)	17	16	40, 57, 114, 140	0
4	C	24/24 (100%)	0.88	2 (8%)	11	10	48, 63, 119, 132	0
5	D	128/128 (100%)	0.93	18 (14%)	2	2	42, 58, 122, 153	0
6	E	22/22 (100%)	1.00	4 (18%)	1	1	48, 63, 107, 164	0
7	F	129/129 (100%)	0.98	22 (17%)	1	1	44, 65, 126, 204	0
8	G	21/21 (100%)	1.54	6 (28%)	0	0	66, 84, 116, 135	0
All	All	616/618 (99%)	0.82	70 (11%)	5	4	37, 59, 119, 204	0

All (70) RSRZ outliers are listed below:

Mol	Chain	Res	Type	RSRZ
7	F	141	HIS	7.6
7	F	5	ASP	7.5
5	D	140	VAL	7.0
8	G	48	THR	6.0
7	F	140	VAL	5.7
3	B	140	VAL	5.5
3	B	141	HIS	5.5
8	G	30	ALA	5.4
5	D	65	PRO	5.3
7	F	142	GLY	5.1
5	D	136	ALA	4.9
6	E	48	THR	4.9
5	D	66	GLU	4.8
5	D	138	ALA	4.8
7	F	51	LEU	4.7
5	D	134	LEU	4.6

Continued on next page...

Continued from previous page...

Mol	Chain	Res	Type	RSRZ
7	F	57	ASN	4.5
3	B	61	HIS	4.3
1	A	59	GLY	4.1
5	D	139	ARG	3.9
7	F	55	LEU	3.9
7	F	137	VAL	3.9
1	A	61	HIS	3.8
5	D	67	ASN	3.8
6	E	49	MET	3.7
7	F	36	ALA	3.7
8	G	32	LEU	3.7
3	B	58	ALA	3.6
4	C	32	LEU	3.4
7	F	81	ALA	3.3
4	C	30	ALA	3.2
5	D	137	VAL	3.1
7	F	70	ILE	3.1
3	B	134	LEU	3.1
6	E	32	LEU	3.1
3	B	60	GLU	3.0
7	F	69	HIS	3.0
5	D	106	ARG	3.0
5	D	68	SER	3.0
7	F	136	ALA	2.9
7	F	130	LEU	2.8
8	G	47	ARG	2.8
7	F	139	ARG	2.7
7	F	7	ILE	2.6
7	F	134	LEU	2.6
5	D	57	ASN	2.6
7	F	40	LEU	2.5
5	D	133	TRP	2.5
7	F	52	LEU	2.4
1	A	95	CYS	2.4
3	B	65	PRO	2.4
8	G	40	LEU	2.4
8	G	29	ALA	2.3
1	A	57	ASN	2.2
1	A	72	ALA	2.2
1	A	89	VAL	2.2
5	D	132	ALA	2.2
1	A	115	VAL	2.2

Continued on next page...

Continued from previous page...

Mol	Chain	Res	Type	RSRZ
6	E	28	SER	2.2
3	B	142	GLY	2.2
5	D	19	ARG	2.2
2	U	40	LEU	2.1
7	F	68	SER	2.1
5	D	135	GLN	2.1
1	A	109	VAL	2.0
5	D	15	ALA	2.0
7	F	47	VAL	2.0
5	D	11	ALA	2.0
7	F	6	ASP	2.0
3	B	67	ASN	2.0

6.2 Non-standard residues in protein, DNA, RNA chains (i)

There are no non-standard protein/DNA/RNA residues in this entry.

6.3 Carbohydrates (i)

There are no monosaccharides in this entry.

6.4 Ligands (i)

In the following table, the Atoms column lists the number of modelled atoms in the group and the number defined in the chemical component dictionary. The B-factors column lists the minimum, median, 95th percentile and maximum values of B factors of atoms in the group. The column labelled 'Q< 0.9' lists the number of atoms with occupancy less than 0.9.

Mol	Type	Chain	Res	Atoms	RSCC	RSR	B-factors($Å^2$)	Q<0.9
10	GOL	A	206	6/?	0.69	0.20	68,90,105,109	0
9	EDO	A	204	4/?	0.71	0.24	62,75,82,87	0
9	EDO	A	205	4/?	0.73	0.20	80,98,116,118	0
9	EDO	F	202	4/?	0.76	0.23	88,106,113,114	0
10	GOL	C	101	6/?	0.83	0.24	75,117,136,141	0
9	EDO	F	201	4/?	0.91	0.14	58,70,75,81	0

6.5 Other polymers (i)

There are no such residues in this entry.

WORLDWIDE PDB PROTEIN DATA BANK

Preliminary Full wwPDB X-ray Structure Validation Report (i)

Jun 30, 2021 – 01:48 pm BST

Deposition ID : D_1292116247

This is a Preliminary Full wwPDB X-ray Structure Validation Report.

This report is produced by the wwPDB Deposition System during initial deposition but before annotation of the structure.

We welcome your comments at *validation@mail.wwpdb.org*
A user guide is available at
https://www.wwpdb.org/validation/2017/XrayValidationReportHelp
with specific help available everywhere you see the (i) symbol.

The following versions of software and data (see references (i)) were used in the production of this report:

MolProbity	:	4.02b-467
Xtriage (Phenix)	:	1.13
EDS	:	2.22
Percentile statistics	:	20191225.v01 (using entries in the PDB archive December 25th 2019)
Refmac	:	5.8.0158
CCP4	:	7.0.044 (Gargrove)
Ideal geometry (proteins)	:	Engh & Huber (2001)
Ideal geometry (DNA, RNA)	:	Parkinson et al. (1996)
Validation Pipeline (wwPDB-VP)	:	2.22

1 Overall quality at a glance (i)

The following experimental techniques were used to determine the structure:
X-RAY DIFFRACTION

The reported resolution of this entry is 2.50 Å.

Percentile scores (ranging between 0-100) for global validation metrics of the entry are shown in the following graphic. The table shows the number of entries on which the scores are based.

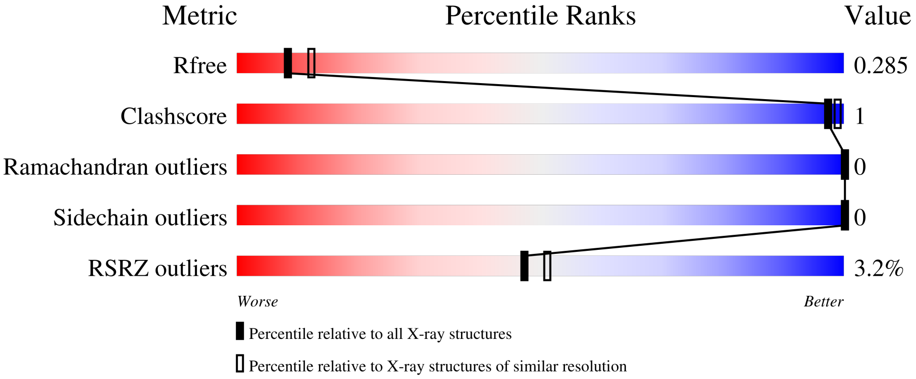

Metric	Whole archive (#Entries)	Similar resolution (#Entries, resolution range(Å))
R_{free}	130704	4661 (2.50-2.50)
Clashscore	141614	5346 (2.50-2.50)
Ramachandran outliers	138981	5231 (2.50-2.50)
Sidechain outliers	138945	5233 (2.50-2.50)
RSRZ outliers	127900	4559 (2.50-2.50)

The table below summarises the geometric issues observed across the polymeric chains and their fit to the electron density. The red, orange, yellow and green segments of the lower bar indicate the fraction of residues that contain outliers for >=3, 2, 1 and 0 types of geometric quality criteria respectively. A grey segment represents the fraction of residues that are not modelled. The numeric value for each fraction is indicated below the corresponding segment, with a dot representing fractions <=5% The upper red bar (where present) indicates the fraction of residues that have poor fit to the electron density. The numeric value is given above the bar.

Mol	Chain	Length	Quality of chain
1	A	137	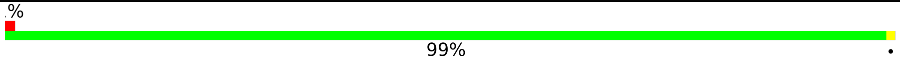
2	U	24	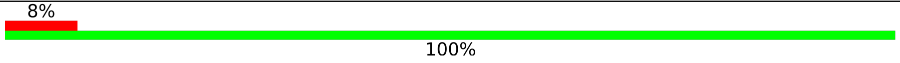
3	B	132	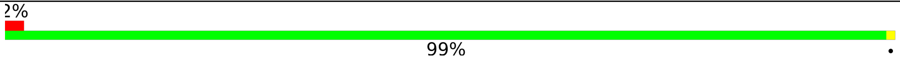
4	C	23	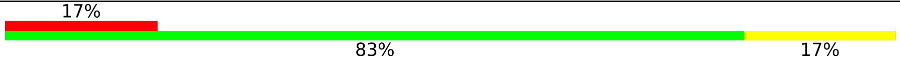

2 Entry composition (i)

There are 5 unique types of molecules in this entry. The entry contains 4946 atoms, of which 2442 are hydrogens and 0 are deuteriums.

In the tables below, the ZeroOcc column contains the number of atoms modelled with zero occupancy, the AltConf column contains the number of residues with at least one atom in alternate conformation and the Trace column contains the number of residues modelled with at most 2 atoms.

- Molecule 1 is a protein.

Mol	Chain	Residues	Atoms						ZeroOcc	AltConf	Trace
			Total	C	H	N	O	S			
1	A	137	2104	657	1046	192	204	5	0	0	0

- Molecule 2 is a protein called GLN-TRP-ALA-ARG-GLU-ILE-GLY-ALA-GLN-LEU-ARG-ARG-MET-ALA-ASP-ASP-LEU-ASN-ALA-GLN-TYR-GLU-ARG-ARG.

Mol	Chain	Residues	Atoms						ZeroOcc	AltConf	Trace
			Total	C	H	N	O	S			
2	U	24	404	124	198	44	37	1	0	0	0

- Molecule 3 is a protein.

Mol	Chain	Residues	Atoms						ZeroOcc	AltConf	Trace
			Total	C	H	N	O	S			
3	B	132	2026	634	1008	183	196	5	0	0	0

- Molecule 4 is a protein called TRP-ALA-ARG-GLU-ILE-GLY-ALA-GLN-LEU-ARG-ARG-MET-ALA-ASP-ASP-LEU-ASN-ALA-GLN-TYR-GLU-ARG-ARG.

Mol	Chain	Residues	Atoms						ZeroOcc	AltConf	Trace
			Total	C	H	N	O	S			
4	C	23	387	119	190	42	35	1	0	0	0

- Molecule 5 is water.

Mol	Chain	Residues	Atoms		ZeroOcc	AltConf
			Total	O		
5	S	25	25	25	0	0

3 Residue-property plots ⓘ

These plots are drawn for all protein, RNA, DNA and oligosaccharide chains in the entry. The first graphic for a chain summarises the proportions of the various outlier classes displayed in the second graphic. The second graphic shows the sequence view annotated by issues in geometry and electron density. Residues are color-coded according to the number of geometric quality criteria for which they contain at least one outlier: green = 0, yellow = 1, orange = 2 and red = 3 or more. A red dot above a residue indicates a poor fit to the electron density (RSRZ > 2). Stretches of 2 or more consecutive residues without any outlier are shown as a green connector. Residues present in the sample, but not in the model, are shown in grey.

- Molecule 1:

Chain A:

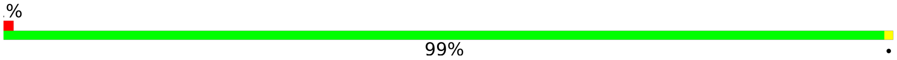

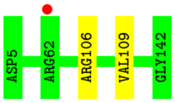

- Molecule 2: GLN-TRP-ALA-ARG-GLU-ILE-GLY-ALA-GLN-LEU-ARG-ARG-MET-ALA-ASP-ASP-LEU-ASN-ALA-GLN-TYR-GLU-ARG-ARG

Chain U:

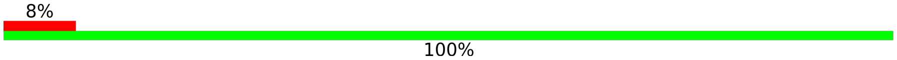

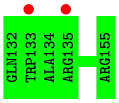

- Molecule 3:

Chain B:

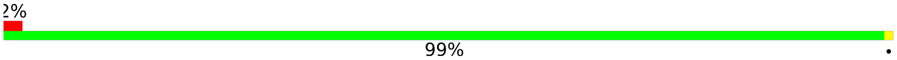

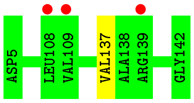

- Molecule 4: TRP-ALA-ARG-GLU-ILE-GLY-ALA-GLN-LEU-ARG-ARG-MET-ALA-ASP-ASP-LEU-ASN-ALA-GLN-TYR-GLU-ARG-ARG

Chain C:

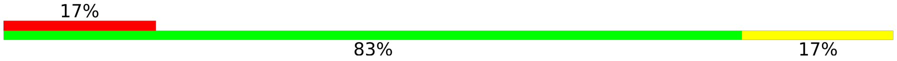

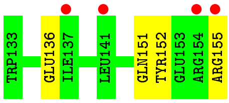

4 Data and refinement statistics (i)

Property	Value	Source
Space group	P 21 21 21	Depositor
Cell constants<br>a, b, c, α, β, γ	49.03Å 69.56Å 92.12Å<br>90.00° 90.00° 90.00°	Depositor
Resolution (Å)	43.28 – 2.50<br>43.28 – 2.50	Depositor<br>EDS
% Data completeness<br>(in resolution range)	97.0 (43.28-2.50)<br>97.0 (43.28-2.50)	Depositor<br>EDS
R_{merge}	(Not available)	Depositor
R_{sym}	(Not available)	Depositor
$< I/\sigma(I) >$ [1]	1.17 (at 2.51Å)	Xtriage
Refinement program	phenix.refine 1.11.1_2575, PHENIX 1.11.1_2575	Depositor
R, R_{free}	0.245 , 0.286<br>0.245 , 0.285	Depositor<br>DCC
R_{free} test set	554 reflections (5.03%)	wwPDB-VP
Wilson B-factor (Å^2)	63.0	Xtriage
Anisotropy	0.285	Xtriage
Bulk solvent k_{sol}(e/Å^3), B_{sol}(Å^2)	0.35 , 50.6	EDS
L-test for twinning[2]	$< \|L\| > = 0.51$, $< L^2 > = 0.34$	Xtriage
Estimated twinning fraction	No twinning to report.	Xtriage
F_o,F_c correlation	0.94	EDS
Total number of atoms	4946	wwPDB-VP
Average B, all atoms (Å^2)	90.0	wwPDB-VP

Xtriage's analysis on translational NCS is as follows: *The largest off-origin peak in the Patterson function is 11.46% of the height of the origin peak. No significant pseudotranslation is detected.*

[1] Intensities estimated from amplitudes.

[2] Theoretical values of $< |L| >$, $< L^2 >$ for acentric reflections are 0.5, 0.333 respectively for untwinned datasets, and 0.375, 0.2 for perfectly twinned datasets.

5 Model quality (i)

5.1 Standard geometry (i)

The Z score for a bond length (or angle) is the number of standard deviations the observed value is removed from the expected value. A bond length (or angle) with $|Z| > 5$ is considered an outlier worth inspection. RMSZ is the root-mean-square of all Z scores of the bond lengths (or angles).

Mol	Chain	Bond lengths		Bond angles	
		RMSZ	#|Z| >5	RMSZ	#|Z| >5
1	A	0.24	0/1075	0.41	0/1461
2	U	0.22	0/208	0.40	0/277
3	B	0.24	0/1033	0.39	0/1402
4	C	0.22	0/199	0.41	0/265
All	All	0.23	0/2515	0.40	0/3405

There are no bond length outliers.

There are no bond angle outliers.

There are no chirality outliers.

There are no planarity outliers.

5.2 Too-close contacts (i)

In the following table, the Non-H and H(model) columns list the number of non-hydrogen atoms and hydrogen atoms in the chain respectively. The H(added) column lists the number of hydrogen atoms added and optimized by MolProbity. The Clashes column lists the number of clashes within the asymmetric unit, whereas Symm-Clashes lists symmetry-related clashes.

Mol	Chain	Non-H	H(model)	H(added)	Clashes	Symm-Clashes
1	A	1058	1046	1046	1	0
2	U	206	198	198	0	0
3	B	1018	1008	1008	1	0
4	C	197	190	190	3	0
5	S	25	0	0	0	0
All	All	2504	2442	2442	4	0

The all-atom clashscore is defined as the number of clashes found per 1000 atoms (including hydrogen atoms). The all-atom clashscore for this structure is 1.

All (4) close contacts within the same asymmetric unit are listed below, sorted by their clash magnitude.

Atom-1	Atom-2	Interatomic distance (Å)	Clash overlap (Å)
4:C:151:GLN:O	4:C:155:ARG:NH1	2.31	0.64
4:C:136:GLU:N	4:C:136:GLU:OE1	2.37	0.56
3:B:137:VAL:HG22	4:C:152:TYR:CG	2.55	0.41
1:A:106:ARG:O	1:A:109:VAL:HG22	2.21	0.41

There are no symmetry-related clashes.

5.3 Torsion angles (i)

5.3.1 Protein backbone (i)

In the following table, the Percentiles column shows the percent Ramachandran outliers of the chain as a percentile score with respect to all X-ray entries followed by that with respect to entries of similar resolution.

The Analysed column shows the number of residues for which the backbone conformation was analysed, and the total number of residues.

Mol	Chain	Analysed	Favoured	Allowed	Outliers	Percentiles	
1	A	133/137 (97%)	132 (99%)	1 (1%)	0	100	100
2	U	22/24 (92%)	21 (96%)	1 (4%)	0	100	100
3	B	126/132 (96%)	125 (99%)	1 (1%)	0	100	100
4	C	21/23 (91%)	21 (100%)	0	0	100	100
All	All	302/316 (96%)	299 (99%)	3 (1%)	0	100	100

There are no Ramachandran outliers to report.

5.3.2 Protein sidechains (i)

In the following table, the Percentiles column shows the percent sidechain outliers of the chain as a percentile score with respect to all X-ray entries followed by that with respect to entries of similar resolution.

The Analysed column shows the number of residues for which the sidechain conformation was analysed, and the total number of residues.

Mol	Chain	Analysed	Rotameric	Outliers	Percentiles	
1	A	110/110 (100%)	110 (100%)	0	100	100
2	U	19/19 (100%)	19 (100%)	0	100	100

Continued on next page...

Continued from previous page...

Mol	Chain	Analysed	Rotameric	Outliers	Percentiles	
3	B	106/106 (100%)	106 (100%)	0	100	100
4	C	18/18 (100%)	18 (100%)	0	100	100
All	All	253/253 (100%)	253 (100%)	0	100	100

There are no protein residues with a non-rotameric sidechain to report.

Sometimes sidechains can be flipped to improve hydrogen bonding and reduce clashes. There are no such sidechains identified.

5.3.3 RNA (i)

There are no RNA molecules in this entry.

5.4 Non-standard residues in protein, DNA, RNA chains (i)

There are no non-standard protein/DNA/RNA residues in this entry.

5.5 Carbohydrates (i)

There are no monosaccharides in this entry.

5.6 Ligand geometry (i)

There are no ligands in this entry.

5.7 Other polymers (i)

There are no such residues in this entry.

5.8 Polymer linkage issues (i)

The following chains have linkage breaks:

Mol	Chain	Number of breaks
3	B	2
1	A	1

All chain breaks are listed below:

Model	Chain	Residue-1	Atom-1	Residue-2	Atom-2	Distance (Å)
1	B	61:HIS	C	67:ASN	N	5.41
1	B	80:GLU	C	82:PRO	N	3.15
1	A	80:GLU	C	82:PRO	N	3.05

6 Fit of model and data (i)

6.1 Protein, DNA and RNA chains (i)

In the following table, the column labelled '#RSRZ> 2' contains the number (and percentage) of RSRZ outliers, followed by percent RSRZ outliers for the chain as percentile scores relative to all X-ray entries and entries of similar resolution. The OWAB column contains the minimum, median, 95th percentile and maximum values of the occupancy-weighted average B-factor per residue. The column labelled 'Q< 0.9' lists the number of (and percentage) of residues with an average occupancy less than 0.9.

Mol	Chain	Analysed	<RSRZ>	#RSRZ>2			OWAB($Å^2$)	Q<0.9
1	A	137/137 (100%)	0.32	1 (0%)	87	89	46, 70, 131, 207	0
2	U	24/24 (100%)	0.35	2 (8%)	11	11	58, 76, 134, 156	0
3	B	132/132 (100%)	0.30	3 (2%)	60	63	48, 78, 124, 154	0
4	C	23/23 (100%)	0.86	4 (17%)	1	1	68, 91, 135, 166	0
All	All	316/316 (100%)	0.35	10 (3%)	47	51	46, 75, 134, 207	0

All (10) RSRZ outliers are listed below:

Mol	Chain	Res	Type	RSRZ
4	C	154	ARG	7.5
1	A	62	ARG	5.5
4	C	155	ARG	4.6
2	U	133	TRP	3.8
3	B	109	VAL	3.5
3	B	108	LEU	3.2
3	B	139	ARG	2.6
2	U	135	ARG	2.3
4	C	141	LEU	2.3
4	C	137	ILE	2.0

6.2 Non-standard residues in protein, DNA, RNA chains (i)

There are no non-standard protein/DNA/RNA residues in this entry.

6.3 Carbohydrates (i)

There are no monosaccharides in this entry.

6.4 Ligands (i)

There are no ligands in this entry.

6.5 Other polymers (i)

There are no such residues in this entry.
